# Exploring the Impact of Positive Psychology-Based Virtual Music Therapy on Mental Health in Stressed College Students during COVID-19: A Pilot Investigation

**DOI:** 10.3390/healthcare12151467

**Published:** 2024-07-23

**Authors:** Jinwoo Han, Hyejin Lee, Teri Kim, Sangyeol Lee

**Affiliations:** 1 Department of Public Health, Graduate School, Wonkwang University, Iksan 54538, Republic of Korea; hanjw.stress@gmail.com (J.H.); stcaecil@hanmail.net (H.L.); 2 Department of Arts Therapy, Daegu Catholic University, Gyeongsan 38430, Republic of Korea; 3 Division of Health and Sport Science, Dongguk University-WISE, Gyeongju 38066, Republic of Korea; terikim@dongguk.ac.kr; 4 Department of Psychiatry, School of Medicine, Wonkwang University, Iksan 54538, Republic of Korea

**Keywords:** virtual music therapy, positive psychotherapy, mental health, stress reduction, anxiety, depression, self-efficacy, resilience, COVID-19

## Abstract

This study explored the effectiveness of a virtual music therapy program, based on positive psychotherapy principles, in college students during the COVID-19 pandemic. Twenty-four undergraduate students with partial PTSD were initially assigned to either an experimental group or a control group, with 11 participants in each group by the study’s end. The experimental group underwent 15 video sessions of the therapy program, completing one session per weekday over 3 weeks. The program involved worksheets targeting goals aligned with positive psychology, such as positive affect, life meaning, personal strengths, gratitude, hope, and happiness. The activities included writing music autobiographies, creating and analyzing song lyrics, and exploring various music pieces. The effectiveness of the intervention was measured using the 21-item Depression Anxiety Stress Scale and the Korean Version of Positive Psychological Capital (K-PPC) before, immediately after, and 3 weeks post-program. The experimental group showed significant improvements in stress (F = 5.759, *p* < 0.05), anxiety (F = 4.790, *p* < 0.01), depression (F = 5.740, *p* < 0.01), self-efficacy (F = 3.723, *p* < 0.05), resilience (F = 4.739, *p* < 0.05), and the K-PPC total score (F = 3.740, *p* < 0.05) compared with the control group. These improvements were maintained at the 3-week follow-up. The findings suggest that positive psychology-based virtual music therapy can significantly enhance the mental health of highly stressed college students, especially during challenging times such as the COVID-19 pandemic.

## 1. Introduction

The global crisis of coronavirus disease 2019 (COVID-19) has had a profound impact on human lives, causing numerous infections and deaths [1]. The uncertainty surrounding the situation and the experience of quarantine have adversely affected people’s mental health. During the COVID-19 pandemic, the prevalence rates of various mental health issues, including depression, anxiety, stress, sleep problems, and overall psychological distress, were observed to be higher in the general population [2,3]. Resulting from the COVID-19 situation, individuals were required to develop coping skills to respond to the sudden changes, such as isolation, infection anxiety, employment restrictions, telecommuting, reduced working hours, and economic impacts [4]. Studies have shown that the pandemic has exacerbated existing mental health conditions and triggered new ones across diverse populations. A systematic review reported increased rates of anxiety, depression, and stress-related symptoms, with healthcare workers, young adults, and individuals with pre-existing mental health conditions being particularly vulnerable [5]. The prevalence of insomnia and sleep disturbances surged during the pandemic, further contributing to the overall psychological distress [6]. The economic consequences, including job losses and financial instability, have been identified as key stressors amplifying mental health problems. Frontline healthcare workers experienced significant psychological distress, including burnout and PTSD symptoms, due to prolonged exposure to high-risk environments and the moral dilemmas posed by resource limitations [7]. Furthermore, research suggests that infectious disease outbreaks can induce stress and contribute to post-traumatic stress disorder (PTSD) symptoms not only in directly infected individuals but also in uninfected populations. For example, a study by Brooks et al. explored the psychological impact of quarantine and isolation measures during infectious disease outbreaks, including COVID-19 [8]. They found elevated levels of stress, anxiety, and PTSD symptoms among individuals regardless of their direct infection status. This underscores the broad-reaching psychological effects of infectious disease outbreaks on mental health.

While the COVID-19 pandemic negatively affected mental health across genders, ages, and races, this study specifically targeted college students undergoing a crucial transition from adolescence to young adulthood, marked by a newfound independence. During this phase, they cultivate diverse relationships, pursue academic goals, and reflect on their identities and career paths. However, this period also correlates with an increased prevalence of mental disorders, such as emotional distress, anxiety, and substance use [9]. Changes in educational and employment landscapes have exacerbated challenges related to identity formation, emotional turmoil, and financial strain [10]. Consequently, stress, anxiety, depression, alcohol consumption, and drug use have escalated among college students during the pandemic, underscoring the urgent need for support and intervention [10,11,12].

To address the mental health challenges among college students, promoting positive factors such as self-efficacy, optimism, hope, and resilience is crucial. Positive psychotherapy, a method rooted in character strengths, emphasizes positive affect [13]. Positive affect, which is a positive factor in personal development and achievement, encompasses inner joy, physical and mental well-being, satisfaction, life balance, and practical aspects that influence human thoughts, emotions, and behavior [14]. Studies have suggested that a higher positive affect is associated with reduced stress, anxiety, and depression, as well as a higher quality of life in college students during the COVID-19 pandemic [15,16]. Positive affect is linked to positive thinking processes, which involve looking at difficult situations in a favorable light and thinking positively about one’s own characteristics and current emotional states [17]. Increased positive affect and positive rumination contribute to a clearer emotional awareness, while employing positive affect as a coping strategy leads to a decreased experience of negative affect in daily life events [17]. This suggests that the principles of positive psychotherapy can serve as effective coping strategies for improving mental health.

To foster resilience and well-being amidst the profound mental health challenges wrought by the COVID-19 pandemic, there arises a pressing need for interventions grounded in positive psychotherapy. In particular, positive psychology-based music therapy is an intervention based on the principles of positive psychotherapy developed by Martin Seligman, aiming to improve an individual’s positive affect, such as strengths, gratitude, and hope, while fostering a deeper sense of meaning in life and the pursuit of overall happiness [18]. In addition, positive psychology-based therapy recognizes the significance of social connections, where individuals can obtain intellectual and emotional satisfaction in their relationships with others as an essential element for a happy life along with personal growth [19]. Recently, there has been a steady increase in research on positive psychology-based music therapy in various populations, such as infants, children, adolescents, patients, and the older adults [20,21,22]. By incorporating activities such as singing, playing instruments, listening to music, and writing lyrics, this approach enables clients to comfortably and authentically engage in therapeutic experiences [23]. Through this process, they can cognitively identify their problems, deal with related emotions, and develop new perspectives for applying and solving psychological problems.

Virtual music therapy emerges as a promising avenue for intervention, harnessing the therapeutic potential of music to cultivate positive emotions, enhance coping mechanisms, and facilitate psychological growth [24]. Furthermore, given the pandemic’s constraints on face-to-face services and the limited mental health personnel, addressing individual psychological difficulties consistently poses significant challenges [25]. In this context, the digital realm affords unprecedented opportunities for the remote delivery of music therapy interventions, enabling individuals to access therapeutic resources from the confines of their homes.

Recently, the effectiveness of virtual music therapy as a coping strategy for mental health during the COVID-19 pandemic has been reported. For example, clinical staff working with patients with COVID-19 participated in a remote receptive music therapy intervention over a 5-week period and indicated a significant decrease in the intensity of tiredness, sadness, fear, and worry [26]. A study that conducted a 12-day program of home-based music therapy in children with developmental delay reported a significant improvement in children’s sleep quality and a reduction in parental distress [26]. However, existing studies on music therapy during the COVID-19 pandemic have certain limitations. These studies typically offered one-on-one personalized therapy sessions, limited experiences to passive listening to music, and primarily focused on clinical populations [26,27]. Consequently, there remains a gap in research regarding the impact of virtual music therapy on addressing the psychological challenges faced by healthy young adults during the pandemic. Furthermore, in the previous studies of music therapy, the main activity has been listening to specific music, which limited participants’ cognitive efforts to actively identify and overcome their situation or problem.

Therefore, this study endeavored to address this gap by developing a self-administered virtual music therapy program grounded in positive psychology principles. The aim of the present study was to investigate the program’s effects on stress, anxiety, depression, and positive affect in college students during the COVID-19 pandemic. By integrating the principles of positive psychology and music therapy into a digital platform, the study aimed to assess its effectiveness in providing collegiate participants with a comprehensive approach to mental health, empowering individuals to navigate the challenges of the pandemic with resilience and optimism. 

## 2. Materials and Methods

### 2.1. Participants

**Figure 1:**
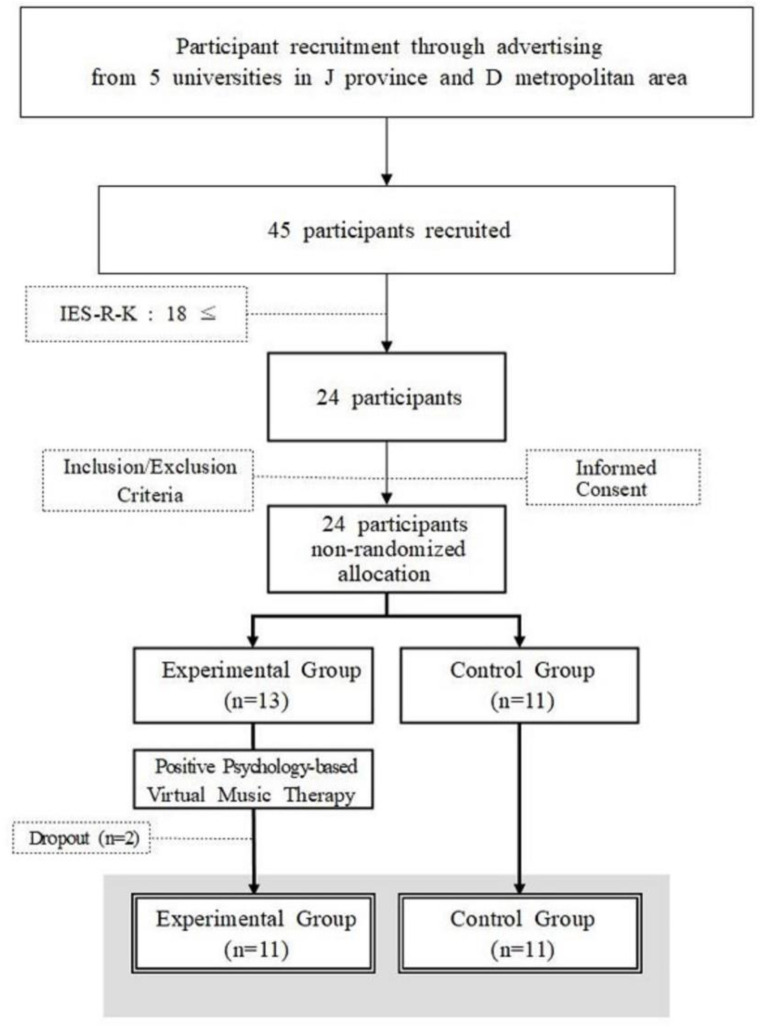
CONSORT flow diagram of participant allocation and progression.

Participants were recruited from four universities located in the J province and one university located in the D metropolitan area in South Korea. To achieve the desired power of the study, calculated using the software G*Power 3.1.9.7, the sample size was determined based on an effect size of 0.50, a significance level of 0.05, and a power of 0.75. The minimum number of participants required was found to be 11 for the experimental group and 11 for the control group, totaling 22 participants. The inclusion criteria for this study included college students aged 20 to 29 years enrolled in a regular undergraduate program during the study period. Participants were required to score higher than 18 points on the Impact of Event Scale-Revised Korean version (IES-R-K), indicating partial post-traumatic stress disorder (PTSD) [28]. Additionally, participants needed to demonstrate the ability to commit to the entire duration of the study, attending all the therapy sessions and completing all the assessments. Applicants were excluded if they had undergone psychiatric treatment in the past, were currently receiving psychiatric treatment, or were undergoing any type of psychological counseling, including music therapy. A total of 45 individuals applied to participate in this study, and after screening with the IES-R-K, 24 participants were confirmed. All of these participants met the remaining inclusion and exclusion criteria for the study and were non-randomly assigned to either the experimental group (*n* = 13), participating in the positive psychology-based virtual music therapy program, or the control group (*n* = 11), receiving no treatment. Two participants dropped out of the experimental group during the study, resulting in a final count of 11 participants in both the experimental and control groups (Figure 1). All the participants received a prior explanation of the research, including its purpose, procedures, the positive psychology-based virtual music therapy program, and the expected benefits, and provided written consent. Approval for this study was obtained from the Institutional Review Board for Ethics in Human Research at Wonkwang University (approval no. WKIRB-202102-HR-005). This study’s clinical trial has been registered with the Clinical Research Information Service (CRIS) associated with the WHO International Clinical Trials Registry Platform (ICTRP) (registration number: KCT0009532).

### 2.2. Measures

#### 2.2.1. Impact of Event Scale-Revised Korean Version (IES-R-K)

The Korean Version of the Impact of Event Scale-Revised (IES-R-K) [24], which was adapted from the original version, was employed as an inclusion criterion [29,30]. This scale consists of 22 items that are restructured into six items for intrusion (e.g., intrusive thoughts, nightmares), six for avoidance (e.g., avoidance of reminders of the traumatic event), five for hyperarousal (e.g., heightened startle response), and five for sleep disturbance and emotional numbing symptoms. This scale uses a 5-point Likert scale ranging from 0 (not at all) to 4 (extremely high). The cutoff points for screening for full PTSD and partial PTSD were 24/25 and 17/18, respectively. The overall Cronbach’s α for the items was 0.89.

#### 2.2.2. 21-Item Version of the Depression Anxiety Stress Scale (DASS 21)

To assess changes in depression, anxiety, and stress resulting from participation in the positive psychology-based virtual music therapy program, we used the 21-Item Version of the Depression Anxiety Stress Scale (DASS-21), which was adapted from the original 42-item version [31,32,33]. The scale consists of three subscales: depression, anxiety, and stress, each with seven items. It utilizes a 4-point Likert scale (0 to 3), with subscale scores obtained by doubling the raw scores. For depression, scores of 0–9 were considered normal and 10–13, 14–20, 21–27, and ≥28 were considered as mild, moderate, severe, and extremely severe depression, respectively. For anxiety, scores of 0–7 were considered normal and 8–9, 10–14, 15–19, and ≥20 were as considered mild, moderate, severe, and extremely severe anxiety. For stress, scores of 0–14 were considered normal and 15–18, 19–25, 26–33, and ≥34 were considered as mild, moderate, severe, and >extremely severe stress. The Cronbach’s alpha for the entire scale was 0.926, and for the depression, anxiety, and stress subscales, the Cronbach’s alpha values were 0.81, 0.84, and 0.85, respectively.

#### 2.2.3. Korean Version of the Positive Psychological Capital (K-PPC)

We evaluated the changes in positive psychological resources among participants using the Korean Version of Positive Psychological Capital (K-PPC), adapted from the Positive Psychological Capital (PsyCap) [34,35]. Consisting of 18 items and four sub-factors, the scale assesses an individual’s positive psychological state, characterized by belief in their ability to pursue goals (hope), confidence in their skills (self-efficacy), capacity to rebound from setbacks (resilience), and an optimistic outlook on future outcomes (optimism), based on a 5-point Likert scale. Overall, Cronbach’s α of the scale was 0.93, with the Cronbach’s α of each sub-factor as follows: self-efficacy 0.879, optimism 0.822, hope 0.837, and resilience 0.723.

### 2.3. Procedure

Prior to the first session of the positive psychology-based virtual music therapy program, the purpose of the study was explained to the participants in both the experimental and control groups, and informed consent was obtained. Sociodemographic characteristics, IES-R-K, DASS 21, and K-PPC scores were obtained from both groups. The experimental group participants received 15 sessions’ worth of video clips of the positive psychology-based virtual music therapy program along with accompanying worksheets at the outset of the research, which they could download onto their personal electronic devices. They were instructed to complete one session per weekday over a 3-week period, granting them autonomy to engage in the program without the constraints of time and space. The DASS 21 and K-PPC scores were reassessed in both groups immediately after the 3-week program concluded, as well as at a 3-week follow-up. This study was conducted in two phases. The first phase took place from 17 February to 20 April 2021, during which the pre, post, and follow-up tests were administered. The second phase occurred from 27 May to 8 July 2021, and followed the same procedure as the first phase, with the pre, post, and follow-up tests conducted during this period.

### 2.4. Intervention: Positive Psychology-Based Virtual Music Therapy

Based on the Positive Psychotherapy Clinical Manual and the items of the DASS 21 and K-PPC, we developed a virtual music therapy program incorporating elements of positive psychology, such as positive affect, meaning of life, personal strengths, gratitude, hope, and happiness [13,18,19,20,21]. The validity of the program was confirmed through consultations with a psychiatrist and two music therapists. The program duration was 3 weeks, with sessions held five times per week, totaling 15 sessions. Each session lasted for 20 min, and all sessions were delivered to participants in a pre-recorded video format. Throughout the program, participants were instructed to engage in tasks aligned with the session theme, such as writing down thoughts, emotions, relationships, and reflections, as directed by the therapist in the video.

The structure of each session was as follows: sessions 1–4 focused on the cognitive perspective of self-awareness, sessions 5–8 on the emotional perspective of self-awareness, and sessions 9–15 on exploring the meaning of life and relationships with others. In each session, the consistent elements included the relaxation and presence phase to bring attention to the here and now through breathing and muscle relaxation, the practice and reflection phase to achieve session-specific goals, and finally, the stage of acceptance where the content recognized during the practice and reflection stages is accepted without judgment. 

Music served three purposes in this program. First, music was used to facilitate the exploration of cognition, emotion, and relationships, enhancing the overall experience. Second, it was used as a means for the participants to actively express and reflect on their experiences, fostering insight and self-awareness. Finally, music provided enjoyment, aiding in coping with anxiety or depression, and induced mood changes through aesthetic experiences [13]. 

This program strategically incorporated musical elements including tempo, tonality, melody, musical texture, timbre, and orchestration. Throughout the sessions, a piano melody in a major key with a BPM of 65 and the sound of ocean waves were consistently provided to the participants as part of the theme-related working process. The therapist also provided piano and guitar accompaniments with a steady beat and a root note to create a safe and predictable musical environment. In addition, depending on the theme of each session, third and fourth chords were added during the music appreciation to provide a richer musical experience. In addition to appreciating the music, the program included activities such as music autobiography, creating new lyrics, analyzing song lyrics, and comparing different pieces of music to help participants achieve session-specific goals. The specific structure of the program, including session goals and content based on positive psychotherapy, is detailed in Table 1.

### 2.5. Data Analysis

A homogeneity test between the experimental and control groups was conducted prior to the study using an independent *t*-test. To analyze the differences in stress, anxiety, depression, and positive psychological capital across the groups (experimental and control) and time points (pre-test, post-test, and follow-up), separate repeated-measures analyses of variance (ANOVAs) were conducted on the DASS 21 subfactors (stress, anxiety, and depression) and the K-PPC subfactors (self-efficacy, optimism, hope, resilience, and total score). All the statistical analyses were performed using SPSS 22.0, and an alpha value of 0.05 was set as the significance level.

## 3. Results

### 3.1. Homogeneity Test of Participant Characteristics

The homogeneity test results for the sociodemographic characteristics, IES-R-K, DASS 21, and K-PPC variables between each group are presented in Table 2. There were no statistically significant differences between the two groups in terms of the sociodemographic characteristics for any of the items.

### 3.2. Outcome Measures

#### 3.2.1. DASS 21

**Figure 2:**
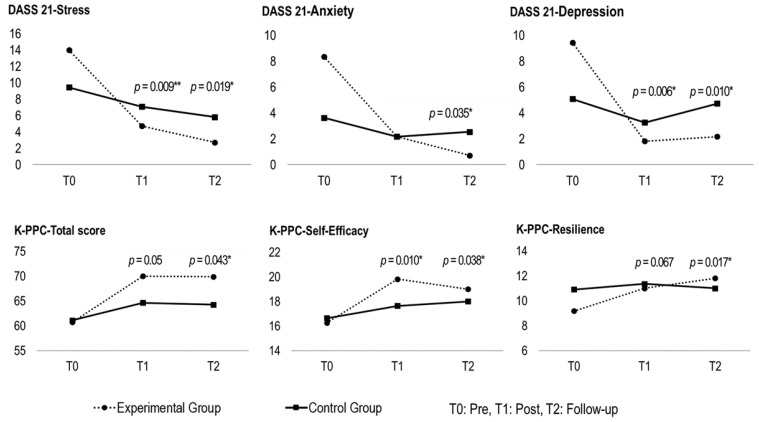
Differences in stress, anxiety, depression, self-efficacy, optimism, hope, and resilience as a result of two (groups: experimental and control) by three (time points: pre-test, post-test, and follow-up) repeated measures ANOVAs. * *p* < 0.05, ** *p* < 0.01.

In the analyses of the DASS 21 as a function of group and time, the statistically significant main effects of time emerged in the stress (F = 19.907, *p* < 0.001), anxiety (F = 9.487, *p* < 0.01), and depression (F = 10.472, *p* < 0.01) variables. In addition, significant group-by-time interaction effects were observed in the stress (F = 5.759, *p* < 0.05), anxiety (F = 4.790, *p* < 0.01), and depression (F = 5.740, *p* < 0.01) variables. The results revealed a significant decrease in stress, anxiety, and depression within the experimental group following the positive psychology-based virtual music therapy, with these improvements sustained at the 3-week follow-up. However, no such changes were observed within the control group (Table 3, Figure 2).

#### 3.2.2. K-PPC

The analyses of the K-PPC as a function of group and time revealed statistically significant main effects of time emerged in self-efficacy (F = 14.381, *p* < 0.001), optimism (F = 3.914, *p* < 0.05), hope (F = 7.317, *p* < 0.01), resilience (F = 6.235, *p* < 0.01), and total score (F = 17.235, *p* < 0.001). Additionally, significant group-by-time interaction effects were found in self-efficacy (F = 3.723, *p* < 0.05), resilience (F = 4.739, *p* < 0.05), and total score (F = 3.740, *p* < 0.05). These results suggest that the positive psychology-based virtual music therapy program effectively enhanced the positive psychological capital of the participants, particularly in terms of self-efficacy and resilience, with these improvements maintained at the 3-week follow-up. However, significant changes were not observed in the control group (see Figure 2 and Table 3).

## 4. Discussion

This study investigated the effects of positive psychology-based virtual music therapy on stress, anxiety, depression, and positive psychological capital (self-efficacy, optimism, hope, and resilience) among college students experiencing high levels of stress during the COVID-19 pandemic. Notably, participants enrolled in the 3-week program experienced significant reductions in stress, anxiety, and depression, coupled with notable enhancements in positive psychological capital, with a particular emphasis on self-efficacy and resilience.

The participants in this study were undergraduate students who showed a tendency for post-traumatic stress disorder on the IES-R-K, indicating their vulnerability to stress. High stress levels in a restricted environment can lead to negative thoughts and emotions and can limit the perspective of looking at current problems in a positive light and thinking about a hopeful future. The students in this study also highlighted the challenges they encountered while adjusting to the educational and technological changes brought about by the COVID-19 pandemic. They expressed feelings of stress and fatigue from coping with the increased volumes of online information and assignments, as well as the considerable time spent in front of computer screens on a daily basis. Consequently, reports of impaired mental health among college students during the COVID-19 era have been consistently documented on a global scale [10,11,12]. Primarily, the observed reductions in stress, anxiety, and depression in the experimental group align with previous research highlighting the therapeutic benefits of music therapy in promoting emotional well-being and reducing psychological distress [36,37,38,39]. In this study, listening to music and participating in diverse musical activities designed to offer enjoyable and meaningful experiences throughout the 15 sessions proved to be effective strategies for coping with negative emotions. In each session, the participants were encouraged to write down their honest feelings and thoughts about the given theme on a worksheet and apply them to their daily lives as actions. Previous research has reported the positive impact of expressive writing on reducing symptoms of depression, anxiety, and stress, thus supporting the effectiveness of the intervention in this study [40].

For college students, heightened depressive symptoms and decreased well-being during the COVID-19 pandemic have been linked to feelings of social isolation and loneliness [41]. In this context, engaging in virtual music therapy sessions may have offered opportunities for participants to connect with the therapist and perceive themselves as part of a collective endeavor with others, even if not physically present. This sense of social connection and belongingness cultivated during the sessions could have served as a protective factor against the negative psychological impacts of social isolation and loneliness. Studies have suggested that practices such as meditation, mindfulness sessions, engaging in phone or online counseling, and participating in digital mental health programs can serve as effective alternatives for alleviating psychological distress and feelings of isolation [42,43].

This study also demonstrated the effectiveness of the positive psychology-based virtual music therapy program in enhancing positive psychological capital, particularly resilience and self-efficacy. Previous studies on the impact of music-based interventions on resilience and self-efficacy have not been abundant. In addition, existing studies present inconsistent findings regarding whether music therapy significantly enhances resilience and self-efficacy, with study participants primarily comprised of clinical populations, particularly children or adolescents [44,45,46,47,48,49,50]. However, it appears that the elements of positive psychology blended into the music therapy program in this study resulted in significant improvements in resilience and efficacy in the undergraduate participants. As presented in Table 1, the program was designed not only to provide relaxation and enjoyment through music but also to enable participants to reflect on past, present, and future events, emotions, and hopes, as well as their personal strengths and relationships through various activities. Such an approach, combining elements of positive psychotherapy with the therapeutic use of music to actively engage participants in activities, might be more conducive to enhancing the overall positive psychological capital, including resilience and self-efficacy, compared with the traditional receptive music therapy approach, which focuses on the passive experience of listening to or experiencing music. This interpretation can be supported by previous studies that have reported the effects of positive psychotherapy on establishing a psychologically safe environment, increasing social connectedness, raising internal hope, and modifying perceptions of coping strategies, ultimately leading to behavioral changes [18,19,20,21,22,23,24,25,26,27,28,29,30,31]. 

The experimental group participants in this study received 15 sessions’ worth of video clips and worksheets at the outset of the research. They were instructed to complete one session per weekday over a 3-week period, granting them autonomy to choose their preferred time and location to perform specific tasks. This emphasis on self-regulation aligns with the research suggesting that autonomy-supportive environments can bolster resilience and self-efficacy through enhanced motivation and perceived competence [51,52]. According to Deci and Ryan’s self-determination theory, when individuals feel autonomous and self-directed in their actions, they experience higher intrinsic motivation, which fosters greater engagement, persistence, and overall psychological well-being [51]. Furthermore, adhering to a daily routine has a positive impact on psychological resilience [53]. Engaging in any regular activity voluntarily can provide a sense of routine and structure, helping individuals to cope with negative emotions more effectively. Therefore, participating in the virtual music therapy program as a structured daily routine might have promoted feelings of control and predictability, thereby increasing resilience and self-efficacy.

The present study has several limitations. There may be selection bias along with the limitation of a small sample size. Excluding individuals who have a history of psychiatric treatment or are currently undergoing psychiatric or psychological counseling introduces potential selection bias in this study. By excluding these participants, the study may inadvertently sample individuals who are generally healthier or less prone to severe mental health issues compared with the broader college student population. Moreover, participants who voluntarily chose to be in the experimental group may have a heightened interest in music therapy or mental health concerns. This self-selection could influence their responses and outcomes, as their motivation and engagement with the therapy may differ from those who did not choose to participate in the intervention. Furthermore, since the intervention delivered music therapy content to participants through pre-recorded video files, it may have been somewhat limited in terms of its therapeutic effectiveness due to communication constraints, such as the therapist’s inability to directly interact with and offer immediate feedback to the client. Therefore, future research should examine how the impacts of positive psychology-based music therapy on mental health differ from the outcomes of this study when delivered in a virtual environment where therapists and clients can interact in real time. In this study, participants were given the autonomy to engage in the program at their preferred time and location. However, despite significant improvement, it is unclear whether this autonomy played a role in enhancing (or diminishing) the effectiveness of the intervention itself. Future research could provide stronger evidence by comparing outcomes when participants have autonomy versus when they adhere to a researcher-determined schedule. Additionally, while worksheets were utilized in the program, their contents were not analyzed or provided with feedback in this study. Incorporating a qualitative analysis of the worksheet data in future research would yield valuable insights. 

In summary, this study delved into the effects of positive psychology-based virtual music therapy on stress, anxiety, depression, and positive psychological capital among college students amidst the backdrop of the COVID-19 pandemic. The results illuminated significant reductions in stress, anxiety, and depression alongside notable enhancements in positive psychological capital, particularly in terms of self-efficacy and resilience among participants. These findings are significant given the documented challenges faced by college students during the pandemic, including increased stress levels and mental health issues. This study underscores the potential of positive psychology-based virtual music therapy as a scalable intervention for addressing mental health challenges among college students, particularly in the context of the COVID-19 pandemic, by leveraging technology and evidence-based therapeutic approaches.

## 5. Conclusions

The findings of the present study suggest the potential of positive psychology-based virtual music therapy as a promising and scalable intervention for addressing stress, anxiety, and depression, and enhancing positive psychological resources among college students. By leveraging technology and evidence-based therapeutic approaches, such interventions have the potential to empower individuals to cultivate resilience and well-being, particularly in challenging circumstances such as the ongoing COVID-19 pandemic. The scalability of this approach is further facilitated by the nature of the program, which allows participants to choose their preferred time and place of participation in a virtual setting. This flexibility may enhance accessibility and effectiveness, making it a valuable tool for promoting mental health in diverse populations. The observed positive outcomes align with previous research highlighting the therapeutic benefits of music therapy in promoting emotional well-being. Moreover, the integration of expressive writing and virtual social connection within the therapy sessions contributed to the effectiveness of the intervention. The emphasis on positive psychology principles, combined with the therapeutic use of music, proved instrumental in fostering resilience and self-efficacy among participants, a notable contribution given the limited research in this area.

## Figures and Tables

**Table 1:** Structure of the positive psychology-based virtual music therapy program.

Elements of Positive Psychotherapy		Positive Psychology-Based Virtual Music Therapy		
Session	Element	Theme	Focus	Contents
1	Positive Introduction and Gratitude Journal	Self-awareness and introduction	Cognitive understanding of myself	Write a music autobiography.
2	Character and Signature Strengths	Finding my strengths		Select strength keywords and match them with my music autobiography.
3	Practical Wisdom	Using my strengths		Write advice for a given story using my strengths.
4	A Better Version of Me	The future of me who has grown up		Write a message (lyrics) to my future self to a rap beat.
5	Open and Closed Memories	Encounter with emotions	Affective understanding of myself	After listening to a song in two different versions (major and minor), discuss the feelings they evoke.
6	Forgiveness	A tolerant attitude		Reflect on others’ mistakes towards me and my emotions, considering forgiveness.
7	Maximizing vs. Satisficing	A fulfilling life		Talk about music that brings me satisfaction and the elements of a fulfilling life.
8	Gratitude	Expressing gratitude		Write a letter expressing gratitude to someone I am grateful for.
9	Hope and Optimism	Door of Hope	The meaning of life and relationship with others	Discuss experiences of finding hope in despair and my expectations for the future.
10	Post-traumatic Growth	To grow beyond pain		Listen to songs about overcoming pain and growth, and reflect on their meanings.
11	Slowness and Savoring	Speed control and mindfulness		Listen to music with a slow tempo to discover thoughts and reflect on slowness in life.
12	Positive Relationships	Positively connected you and me		Find the strengths of the people around me and write instruments and lyrics for the music.
13	Positive Communication	Communication with positivity		Write a positive letter to someone with concerns.
14	Altruism	Compassion		Recall misunderstandings with others, past gifts, and associated emotions.
15	Meaning and Purpose	The meaning and purpose of life		Write a letter to myself reflecting on aspirations, desired legacy, and how I want to be remembered.

The elements of positive psychotherapy correspond to the session-based program components outlined in the Positive Psychotherapy Clinician Manual by Tayyab Rashid and Martin Seligman [13]. Based on this framework, the themes, focus, and contents of Positive Psychology-based Virtual Music Therapy were developed through consultation with a psychiatrist and two professional music therapists.

**Table 2:** Homogeneity test results for the sociodemographic characteristics and pre-tested outcome measures.

Variables		Experimental Group (N = 11)	Control Group (N = 11)	*t*/*Χ*^2^	*p*
Age (years)	M ± SD	21.18 ± 1.47	21.36 ± 2.25	−0.224	0.825
Sex (N, %)	Male	1 (9.1)	1 (9.1)	0.000	1.000
	Female	10 (90.9)	10 (90.9)		
Academic Year (N, %)	First	0 (0)	2 (18.2)	4.467	0.215
	Second	2 (18.2)	1 (9.1)		
	Third	4 (36.4)	1 (9.1)		
	Fourth	5 (45.4)	7 (63.6)		
Religion (N, %)	Christianity	9 (81.8)	5 (45.5)	03.429	0.180
	Buddhism	0 (0)	1 (9)		
	None	2 (18.2)	5 (45.5)		
Sleeping Time (hour)	M ± SD	5.96 ± 1.46	6.36 ± 1.34	−0.685	0.501
IES-R-K		36.55 ± 13.02	32.18 ± 6.21	1.003	0.332
DASS 21	Stress	14.00 ± 8.94	9.45 ± 6.07	1.394	0.181
	Anxiety	8.36 ± 8.66	3.64 ± 2.94	1.714	0.102
	Depression	9.45 ± 6.46	5.09 ± 4.13	1.888	0.076
K-PPC	Total	60.73 ± 9.34	61.09 ± 9.09	−0.093	0.927
	Self-Efficacy	16.27 ± 2.97	16.64 ± 2.84	−0.294	0.772
	Optimism	17.73 ± 2.90	16.55 ± 3.14	0.916	0.370
	Hope	17.55 ± 2.54	17.00 ± 3.41	0.426	0.675
	Resilience	9.18 ± 3.06	10.91 ± 2.47	−1.457	0.161

To verify the homogeneity between the two groups, independent sample *t*-tests were conducted for age, sleeping time, IES-R-K, DASS 21, and K-PPC. Chi-square tests were used for sex, academic year, and religion. N, number of participants; M, Mean; SD, standard deviation; IES-R-K, the Impact of Event Scale-Revised Korean Version; DASS 21, the 21-item version of the Depression Anxiety Stress Scale; K-PPC, the Korean version of positive psychological capital.

**Table 3:** Comparisons of the DASS 21 and K-PPC as a function of group and time using repeated measures ANOVAs.

Variables			Experimental Group (N = 11)	Control Group (N = 11)	Group	Time	Group by Time
			M ± SD	M ± SD	F	F	
DASS 21	Stress	pre	14.00 ± 8.94	9.45 ± 6.07	0.018	19.907 ***	5.759 *
		post	4.73 ± 3.26	7.09 ± 6.28			
		follow-up	2.73 ± 3.50	5.82 ± 7.67			
	Anxiety	pre	8.36 ± 8.66	3.64 ± 2.94	0.460	9.487 **	4.790 *
		post	2.18 ± 1.89	2.18 ± 3.28			
		follow-up	0.73 ± 1.35	2.55 ± 4.48			
	Depression	pre	9.45 ± 6.46	5.09 ± 4.13	0.005	10.472 **	5.740 *
		post	1.82 ± 3.63	3.27 ± 4.13			
		follow-up	2.18 ± 3.40	4.73 ± 7.00			
K-PPC	Total	pre	60.73 ± 9.34	61.09 ± 9.09	0.920	17.235 ***	3.740 *
		post	70.00 ± 8.80	64.64 ± 9.12			
		follow-up	69.91 ± 8.25	64.27 ± 10.95			
	Self-efficacy	pre	16.27 ± 2.97	16.64 ± 2.84	0.643	14.381 ***	3.723 *
		post	19.82 ± 3.34	17.64 ± 2.50			
		follow-up	19.00 ± 2.49	18.00 ± 3.80			
	Optimism	pre	17.73 ± 2.90	16.55 ± 3.14	2.200	3.914 *	0.688
		post	19.64 ± 2.54	17.91 ± 3.75			
		follow-up	19.18 ± 2.75	16.64 ± 4.27			
	Hope	pre	17.55 ± 2.54	17.00 ± 3.41	1.420	7.317 **	0.714
		post	19.55 ± 3.30	17.73 ± 2.20			
		follow-up	19.91 ± 1.92	18.64 ± 3.04			
	Resilience	pre	9.18 ± 3.06	10.91 ± 2.47	0.192	6.235 **	4.739 *
		post	11.00 ± 2.68	11.36 ± 2.01			
		follow-up	11.82 ± 2.48	11.00 ± 2.37			

N, number of participants; M, Mean; SD, standard deviation; DASS 21, the 21-item version of the Depression Anxiety Stress Scale; K-PPC, the Korean version of positive psychological capital. * *p* < 0.05, ** *p* < 0.01, *** *p* < 0.001.

## Data Availability

The data presented in this study are openly available in Google Docs at [https://docs.google.com/spreadsheets/d/1pixJV6lNkXIDnvRabgnCMF1pxW1wU9z1/edit?usp=sharing&ouid=115163458442950089664&rtpof=true&sd=true], accessed on 20 July 2024.
